# Absence of arrhythmogenic effects in cAMP subcellular compartments supports PDE8 as a target in atrial fibrillation

**DOI:** 10.1016/j.jmccpl.2026.100871

**Published:** 2026-09-26

**Authors:** David Revuelta, Nefeli Grammatika Pavlidou, Cristina Aceituno, Issam Abu Taha, Andrea Brunetti, Antonino Ginel, Evaldas Girdauskas, Dobromir Dobrev, Leif Hove-Madsen, Cristina E. Molina

**Affiliations:** a Institute of Experimental Cardiovascular Research, University Medical Center Hamburg –Eppendorf (UKE), Hamburg, Germany; b DZHK (German Centre for Cardiovascular Research), partner site North, Germany; c Biomedical Research Institute Barcelona (IIBB-CSIC) and Institut de Recerca Sant Pau (IR Sant Pau), Hospital de la Santa Creu i Sant Pau, Barcelona, Spain; d Institute of Pharmacology, Faculty of Medicine, University Duisburg-Essen, Essen, Germany; e Cardiac Surgery Department and IR Sant Pau, St. Antoni Mª Claret 167, 08025, Barcelona, Spain; f Department of Cardiovascular Surgery, University Heart & Vascular Center Hamburg UKE, Hamburg, Germany; g Department of Integrative Physiology, Baylor College of Medicine, Houston, TX, USA; h Department of Medicine, Montreal Heart Institute and Université de Montréal, Montréal, QC, Canada; i CENTRO DE INVESTIGACIÓN BIOMÉDICA EN RED, Enfermedades Cardiovasculares (CIBERCV), Madrid, Spain

**Keywords:** cAMP, Human atria, PDE8, Atrial fibrillation, Ca^2+^ handling

## Abstract

**Background:**

Targeting Phosphodiesterase 8B (PDE8B) has been proposed as a strategy to restore L-type Ca^2+^ current (I_Ca,L_) in patients with atrial fibrillation (AF). However, the effects of total PDE8 inhibition on other Ca^2+^ handling or 3′,5′-cyclic adenosine monophosphate (cAMP) nanodomains have not been assessed and thus the potential proarrhythmic consequences are still undetermined.

**Objective:**

The aim of this study was to assess whether PDE8 contributes to the regulation of cAMP signaling in other Ca^2+^ handling-related compartments and to sarcoplasmic reticulum Ca^2+^ release-mediated arrhythmogenic events.

**Results:**

Fluorescence resonance energy transfer (FRET) live-cell imaging, confocal Ca^2+^ imaging and co-immunoprecipitation (Co-IP) were performed in atrial myocytes and tissues allocated across experimental assays from a total cumulative cohort of 141 patients with or without AF (sinus rhythm, Ctl). cAMP levels were not significantly increased upon non-isoform-selective total PDE8 inhibition in the cytosol, in ryanodine receptor 2 (RyR2) or in the sarcomere nanodomains, troponin I (TpnI) and myosin binding protein C (MyBP-C). Consistently, no significant changes in the frequency of arrhythmogenic Ca^2+^ sparks or Ca^2+^ waves after total PDE8 inhibitor application were detected.

**Conclusion:**

These results suggest that PDE8 inhibition promotes a compartment-specific regulation of cAMP signaling that exclusively affects the L-type Ca^2+^ channel in human atrial myocytes. Therefore, PDE8 inhibition can be used to restore I_Ca,L_ in patients with AF, without promoting spontaneous arrhythmogenic Ca^2+^ releases in isolated human atrial myocytes.

## Highlights

• PDE8 inhibition does not affect the global levels of cAMP in human atrial myocytes.
• PDE8 inhibition does not regulate cAMP in the sarcomere or in RyR2.
• PDE8A is associated with RyR2 in the human atria.
• PDE8 inhibition does not acutely increase sarcoplasmic reticulum Ca^2+^ release.

## Graphical abstract

**Figure:**
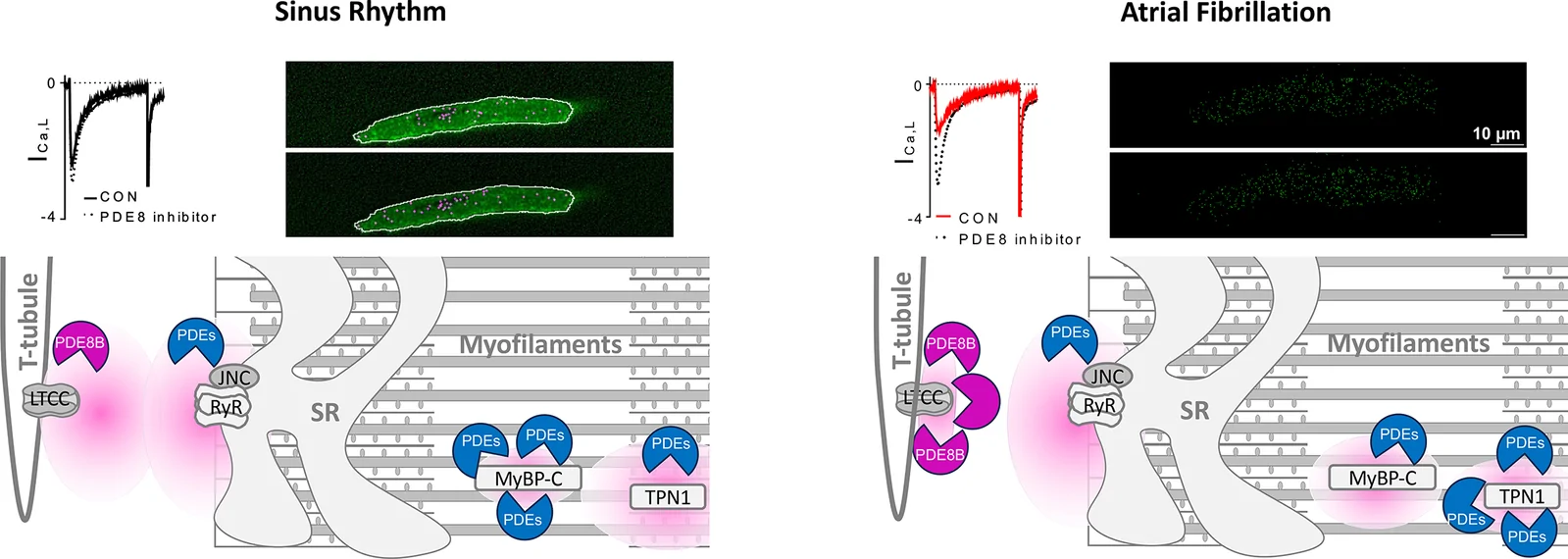

## 1 Introduction

cAMP compartmentation is crucial in the regulation of excitation-contraction coupling [1]. Upon arrival of an action potential, the membrane of the cardiomyocyte depolarizes, triggering the opening of L-type Ca^2+^ channels and generating an inward current of Ca^2+^ from the extracellular space into the cell (I_Ca,L_). This triggers a massive release of the Ca^2+^ stored in the sarcoplasmic reticulum to the cytosol through the RyR2, which activates contraction. In the sarcomere, troponin acts as a Ca^2+^-dependent inhibitory switch blocking the actin binding sites of myosin thick filaments. When troponin binds Ca^2+^, it slides away exposing the actin binding sites and permitting the interaction of myosin and actin filaments that generates the contractile force [1]. Simultaneously, MyBP-C regulates the amount of myosin heads available for interaction, thus modulating contraction force [2]. Each one of these effectors (L-type Ca^2+^ channels, RyR2, troponin and MyBP-C) is individually regulated in a cAMP nanodomain [3], [4], [5], [6], [7], [8], [9], [10], enabling a fine protein kinase A (PKA)-mediated regulation of Ca^2+^ handling and cardiac function.

Our group demonstrated that, in human atrial cardiomyocytes, cAMP degradation is essentially mediated by the classical cardiac phosphodiesterases (PDE1 to 4) both in basal and upon β-adrenergic stimulation [11]. Additionally, we and others have described that alterations in cAMP signaling contribute to the AF-associated remodeling. These alterations include the reduction of the activity [11], regulation [12] or expression of PDEs [13], [14] as well as PKA-dependent hyper-phosphorylation of most of the key proteins for Ca^2+^ handling [14], [15], [16], [17]. However, in persistent AF, there is a paradoxical reduction of I_Ca,L_ mediated by PKA-dependent hypo-phosphorylation [15]. We recently showed that this reduction occurs *via* upregulation of PDE8B [18], which had not been considered as a relevant enzyme in the regulation of cAMP [19], [20] in the heart until then. Moreover, the study demonstrated that PDE8B co-localizes with the L-type Ca^2+^ channel, particularly in persistent AF [18], and PDE8B inhibition resulted in an increase of the sarcolemmal cAMP levels leading to I_Ca,L_ normalization. These findings highlight PDE8B as a novel atrial-specific regulator of atrial arrhythmogenesis. Restoring I_Ca,L_ *via* PDE8B inhibition could therefore be considered as a novel selective treatment for patients with persistent AF. However, PDE8B-selective inhibitors are currently unavailable. Consequently, testing this therapeutic rationale relies on non-isoform-selective total PDE8 inhibitors (such as PF-04957325), which target both PDE8B and PDE8A. This lack of selectivity in the inhibitors poses a potential challenge, as knock-out of PDE8A has been shown to affect the RyR2 complex and increase the frequency of spontaneous sarcoplasmic reticulum Ca^2+^ release in mouse ventricular myocytes, suggesting potential proarrhythmic off-target effects for total PDE8 inhibitors [19].

Therefore, to evaluate the viability of this pharmacological strategy, the aim of the present study is to investigate the acute effects of total PDE8 inhibition in the regulation of other key Ca^2+^ handling nanodomains in human atrial myocytes, with a particular focus on exploring the PDE8A-RyR2 association and the regulation of spontaneous Ca^2+^ release from the sarcoplasmic reticulum.

## 2 Methods

### 2.1 Human atrial tissue

This study was conducted using atrial tissue samples obtained from a total cumulative cohort of 141 patients undergoing heart surgery. Individual tissue samples were allocated across the various experimental sub-assays with exact cell (n) and patient (N) counts for each comparison detailed in Supplementary Table 2A. A comprehensive breakdown of tissue allocation and experimental fate including atrial chamber origin, AF classification, and patient overlap across multiple assays is provided in Supplementary Table 2B. All of the procedures were managed in accordance with the Declaration of Helsinki for experiments involving human samples and all of the patients gave informed consent. This project was approved by the Ethical Board of the Medical Chamber Hamburg (No. WF-088/18) and the Ethics Committee of the Hospital de Sant Pau i la Santa Creu (IIBSP-AMP-2024-213).

Human atrial samples were obtained from discarded tissue during cardiac surgical interventions and directly placed in cold Custodiol ® HTK solution (Dr. Franz Köhler Chemie GmbH, Bensheim, Germany) for transportation. In the laboratory, tissue samples were cleared by separating it from clotted blood and adipose and fibrotic tissue. Cleared tissue was either snap-frozen in liquid nitrogen and kept at −80 °C for co-immunoprecipitation (Co-IP) or immediately subjected to cell isolation.

### 2.2 Co-immunoprecipitation and Western blot analysis

Snap-frozen tissue samples were homogenized in immunoprecipitation buffer containing 10 mM HEPES, 5 mM EDTA, 500 mM Sucrose, 0.1% SDS, 0.5% Triton, 20 mM NaF, 1 mM Na_3_VO_4_, Protease- and Phosphatase-Inhibitor Cocktail. Equal amounts of protein lysate, 500 mg per sample, were incubated overnight with 2 mg anti-RyR2 antibody (Thermo Fisher; MA3–916) and 150 ml Protein G Sepharose beads (GE Healthcare). The resulting RyR2 immunoprecipitates were washed four times with PBS, with centrifugation at 800 x*g* for 2 min at 4 °C after each wash. Bound proteins were eluted by boiling in 2× loading buffer at 95 °C for 5 min. After centrifugation at 800 x*g*, for 2 min at 4 °C, 30 ml of each immunoprecipitated sample were resolved by SDS-PAGE on 8–12% gels and analyzed by Western blotting. Co-immunoprecipitated PDE8A was detected using an anti-PDE8A antibody (Santa Cruz Biotechnology; sc-30,059, rabbit polyclonal, 1:500), whereas RyR2 was detected as immunoprecipitation control using anti-RyR2 antibody (Thermo Fisher Scientific; MA3–916). Near-infrared fluorophore-conjugated secondary antibodies were visualized and quantified using the Odyssey CLx imaging system (LI-COR Biosciences, Lincoln, NE, USA).

### 2.3 Isolation and culture of human atrial myocytes

Cardiomyocytes were isolated using a previously described protocol [11]. Tissue dissociation was conducted at 37 °C in Ca^2+^-free Tyrode's solution (containing in mM: 88 Sucrose, 88 NaCl, 5.4 KCl, 4 NaHCO_3_, 0.3 NaH_2_PO_4_, 1.1 MgCl_2_, 10 HEPES, 20 Taurine, 10 Glucose, 5 sodium pyruvate, pH 7.4). After cleaning, the tissue was cut into fragments of approximately 1 mm^3^. Tissue fragments were then incubated in Ca^2+^-free Tyrode's solution with 0.5 mg/ml collagenase type 2 (240 U/mg; LS004176, Worthington Biochemical, Lakewood, NJ, USA), 0.5 mg/ml proteinase type XXIV (11 U/mg; P8038, Sigma-Aldrich, St. Louis, MO, USA), and 2% bovine serum albumin (BSA; A7906, Sigma-Aldrich, St. Louis, MO, USA) for 30 min at 37 °C and stirring at 250 rpm. Afterwards, the tissue was switched to Ca^2+^-free Tyrode's solution containing 5% BSA and 2 μM blebbistatin (B0560, Sigma-Aldrich, St. Louis, MO, USA). To further facilitate cell disaggregation, gentle agitation with a Pasteur pipette was applied for 5 min. Following agitation, tissue fragments were placed in Ca^2+^-free Tyrode's solution containing 0.4 mg/ml collagenase and 2% BSA and incubated 15 min at 37 °C with stirring at 250 rpm. The digestion/disaggregation cycle was repeated up to 5 times to increase the yield of isolated cells. Finally, cells were collected by centrifugation at 500 rpm for 5 min. Freshly isolated cardiomyocytes were either used for confocal Ca^2+^ imaging or cultured for 48 h to perform FRET live-cell imaging.

For culture, cardiomyocytes were resuspended in M1 culture medium [MEM (M4780; Sigma-Aldrich, St. Louis, MO, USA) supplemented with 2.5% foetal bovine serum (16,000,044; Invitrogen, Carlsbad, CA, USA), 1% penicillin-streptomycin (P4333; Sigma-Aldrich, St. Louis, MO, USA) and 2 μM blebbistatin] and cultured for 2 h at 37 °C and 5% CO_2_ for cell adhesion on laminin (23,017,015; Thermo Fisher Scientific, Waltham, MA, USA)-coated glass-bottom dishes. Afterwards, the medium was changed to M2 (foetal bovine serum-free M1) and the cells were transduced with FRET biosensor-encoding adenoviruses by incorporating them into the medium at a density of 200 PFU/cell. Expression was achieved after 24 to 48 h of culture, depending on the construct.

### 2.4 FRET live cell imaging

Human atrial myocytes were transduced with adenoviral vectors encoding for either cytosolic FRET-based biosensor Epac1-camps [21] or nanodomain-targeted biosensors, Epac1-JNC [22], TpnI-CUTie [23] and MyBPC [10]. For imaging, cells were placed into FRET buffer (containing in mM: 144 NaCl, 5.4 KCl, 1 CaCl_2_, 1 MgCl_2_, 20 HEPES). Cells were visualized in a set-up consisting of a Leica DMI3000 inverted microscope (Leica Microsystems, Wetzlar, Germany), equipped with a sCMOS optiMOS camera (QImaging, Surrey, BC, Canada) and a 63×/1.4 oil immersion objective. The system was coupled with a Dual View beam splitter (Cube 05-EM, 505 dcxr, D480/30 m, D535/40; Photometrics, Tucson, AZ, USA) and a LED excitation source pE-100 (CoolLED, Andover, UK). Viable cells were identified by their characteristic rod shape and striation. Cells were then excited by a single wavelength (440 nm) LED operated by an Arduino microcontroller (Arduino, Ivrea, Italy). Individualization of the donor and acceptor fluorophores signals was conducted by splitting the emission light at 475/30 and 527/40 nm, respectively. Images were taken every 5 s with 5 ms exposure. The time-lapse ratio of both signals was generated using Micro-Manager software version 1.4.5 (Vale Lab, University of California, San Francisco, CA, USA). For display purposes, representative time-course FRET ratio traces were smoothed using a 2nd-order polynomial Savitzky-Golay filter with a 9-point moving window.

### 2.5 Confocal Ca^2+^ imaging

Measurement of cytosolic Ca^2+^ levels was performed using a Leica SP5 AOBS resonant-scanning confocal microscope (Leica Microsystems, Wetzlar, Germany), equipped with a 63× glycerol immersion objective and hybrid detector (HyD). The high affinity Ca^2+^ dye Cal-520 (21,130; AAT Bioquest, Pleasanton, CA, USA) was used to visualize cytosolic Ca^2+^. Myocytes were loaded with 2 μM Cal-520 in phosphate buffer saline (PBS) for 20 min at room temperature, followed by a minimum of 40 min de-esterification period. Cells were excited at 488 nm and the fluorescence emission was collected between 495 and 650 nm at a frame rate of 90 Hz.

To ensure an objective quantification, Ca^2+^ sparks detection and analysis were conducted using a custom-made automated detection algorithm based on a continuous wavelet transform, as previously described [24]. Filter parameters for accepting spark candidates were: relative amplitude (F/F_0_) above 0.25, time to peak 10–200 ms, full duration at half maximum (FDHM) between 10 and 200 ms, decay constant (tau) between 10 and 200 ms, and R^2^ for the fit of the decay above 0.3. For every validated spark, the software calculated spark properties including ΔF/F_0_, tau, full width at half maximum (FWHM) and FDHM. Additionally, the frequency of events through the experiment was normalized to the estimated cell area to determine the spark density. Because macroscopic events, Ca^2+^ waves, could be identified visually, these were directly measured in the recorded image stack using Leica Application Suite X software version 6.1 (Leica Microsystems, Wetzlar, Germany).

### 2.6 Statistical analysis

Statistical Analysis was conducted in RStudio version 2026.01.1 + 403 (Posit Software, Boston, MA, USA). For the analysis of patients' characteristics, the distribution of categorical variables was compared between groups (Ctl and AF) using Fisher's exact test. For continuous variables, normality was assessed through Shapiro-Wilk test and group comparison was performed by unpaired Student's *t*-test (for normally distributed variables) or Wilcoxon's rank test (for non-normally distributed variables).

To account for pseudo-replication in single-cell experiments, continuous data were analyzed using Linear Mixed Models (LMM) with the R package lme4 (v1.1–26)25. Treatment (baseline *vs.* PF) and clinical group (Ctl *vs.* AF) were set as fixed effects. The hierarchical structure of the data was modelled by setting individual cells nested within patients as random intercept effects, accounting for inter-patient variability and the repeated-measures (paired) design of the before-and-after treatment cellular recordings. LMM diagnostics were systematically performed by assessing the normality of the model residuals *via* Shapiro-Wilk testing; variables exhibiting non-normal residuals were log-transformed to satisfy model assumptions The potential confounding impact of clinical characteristics on data variance was screened, and significant variables were adjusted as covariates in the final models, applying a conservative limit (maximum one covariate per 10 patients) to prevent model overfitting. These variables consisted of epidemiological data, atrial chamber origin (right *vs.* left atrium), type of surgical intervention, and pharmacological treatment prior to intervention. Epidemiological data included body mass index (BMI), left ventricular ejection fraction (LVEF), sex and previous diagnostic of diabetes. Surgeries were classified as coronary artery bypass (CAB); valve surgery, including mitral, aortic, or tricuspid repairs and replacements, as well as aortic root reconstructions; and other type of procedures including pulmonary vein isolation, atrial ablation, left atrial appendage closure, ascending aorta surgery, and left ventricle assisting device implantation. Finally, pharmacological treatments were classified as angiotensin converting enzyme (ACE) inhibitors, angiotensin II receptor blockers (ARBs), beta-blockers, Ca^2+^ antagonists, diuretics, nitrates/NO donors, statins, antiarrhythmic drugs and others. Post-hoc multiple comparisons were performed using estimated marginal means (EMMs). To evaluate functional safety and clinical relevance beyond null-hypothesis significance testing, absolute effect sizes, 95% Confidence Intervals (95% CIs) and standardized Effect Sizes (Cohen's *d* with corresponding 95% CIs) were extracted for all EMM comparisons. To control for Type I error inflation due to multiple testing, raw *p*-values were adjusted using False Discovery Rate (FDR) correction according to the Benjamini-Hochberg procedure [25].

For FRET data, a one-sample *t*-test applied to the EMMs was used to determine whether treatment effects significantly deviated from baseline zero. Because Ca^2+^ wave frequency features a zero-inflated distribution, this parameter was strictly analyzed using a Negative Binomial Generalized Linear Mixed Model (GLMM) *via* the *glmmTMB* package, incorporating the logarithm of the recording duration as an offset term to directly model event incidence rates (waves/min). All detailed statistical outputs, including effect sizes, 95% CIs, and FDR-adjusted *p*-values, are reported in Supplementary Table 1.

Normally distributed data are presented as mean ± standard error of the mean (SEM) whereas non-normally distributed data are presented as median and interquartile range (IQR). In all cases statistical significance was determined by a cut-off *p*-value of *p* < 0.05. All graphs were generated using GraphPad Prism version 8.0.2 (GraphPad Software, San Diego, CA, USA).

## 3 Results

### 3.1 Screening of the patients' clinical features

The characteristics of the cohort of patients included in this study (*N* = 141) are detailed in Table 1, with specific sample sizes, clinical features, and tissue allocation for each experimental sub-assay summarized in Supplementary Table 2A and B. Significant differences were found between the Ctl (*N* = 81) and AF (*N* = 60) groups. Particularly, patients included in the AF group were older (70.5 *vs.* 64 years, *p* < 0.001) and underwent predominantly valve surgery (69.5%, *p* = 0.003) and/or other types of procedures (69.5%, *p* < 0.001), whereas tissues from the Ctl group were often obtained from CAB surgeries (65.4%, *p* = 0.015). Furthermore, AF patients used beta-blockers (*p* < 0.001) and diuretics (*p* < 0.001) more frequently.

To prevent these clinical imbalances from acting as confounders in the experimental sub cohorts, covariates were screened and statistically adjusted for in the LMM when they were shown to affect the variance of the experimental data.

**Table 1:** Baseline clinical characteristics of Ctl and AF cohorts of patients. Abbreviations: control-sinus rhythm (Ctl.), atrial fibrillation (AF), body mass index (BMI), left ventricular ejection fraction (LVEF), coronary artery by-pass (CAB), angiotensin-converting enzyme (ACE), angiotensin receptor blockers (ARBs). ‘Other procedures’ include pulmonary vein isolation, atrial ablation, left atrial appendage closure, ascending aorta surgery, and left ventricle assisting device implantation. The ‘Other’ pharmacological treatments category includes antiplatelet agents, anticoagulants, antidiabetic drugs, analgesics (including steroidal and non-steroidal anti-inflammatory drugs), psychiatric medications (antipsychotics, antidepressants, and anxiolytics), proton pump inhibitors, hormone therapies, respiratory drugs, and nutritional supplements.

	Ctl (*n* = 81)	AF (*n* = 60)	*p*-value
Epidemiological characteristics			
Age, years	64.00 [58.00–72.25]	70.50 [65.50–77.00]	0.001
BMI, kg/m^2^	26.56 [24.18–29.28]	26.37 [23.68–29.96]	0.927
LVEF, %	56.50 [50.75–60.00]	55.00 [47.75–60.00]	0.194
Right atrium, n (%)	76 (93.8%)	39 (65.0%)	< 0.001
Sex (male), n (%)	62 (76.5%)	43 (71.7%)	0.561
Diabetes, n (%)	24 (29.6%)	14 (23.3%)	0.447

Surgery			
Valve surgery, n (%)	37 (45.7%)	42 (70.0%)	0.006
CAB, n (%)	51 (63.0%)	27 (45.0%)	0.041
Other procedures n (%)	15 (18.5%)	41 (68.3%)	< 0.001

Pharmacological treatment			
ACE inhibitors, n (%)	34 (42.0%)	24 (40.0%)	0.864
ARBs, n (%)	20 (24.7%)	17 (28.3%)	0.700
Beta-blockers, n (%)	35 (43.2%)	54 (90.0%)	< 0.001
Ca^2+^ antagonists, n (%)	19 (23.5%)	19 (31.7%)	0.338
Diuretics, n (%)	19 (23.5%)	34 (56.7%)	< 0.001
Nitrates, n (%)	1 (1.2%)	5 (8.3%)	0.083
Statins, n (%)	41 (50.6%)	34 (56.7%)	0.499
Antiarrhythmic, n (%)	2 (2.5%)	10 (16.7%)	0.004
Other, n (%)	64 (79.0%)	59 (98.3%)	0.001

### 3.2 PDE8 does not regulate the global levels of cAMP

We initially determined the effect of total PDE8 inhibition on total levels of cAMP in human atrial myocytes. To do so, we used the cytosolic FRET biosensor Epac1-camps and visualized real-time changes in cAMP in living human atrial cardiomyocytes upon total PDE8 inhibition using 30 nM PF-04957325 (PF; PZ0211; Sigma-Aldrich, St. Louis, MO, USA). PF elicited a similar response in both groups (1.07 ± 0.55% in Ctl *vs.* 1.51 ± 0.22% in AF, *p* = 0.670) that was not significantly different from zero (*p* = 0.454 in Ctl; *p* = 0.750 in AF) (Fig. 1). Furthermore, the addition of PF did not further enhance the response of 3-isobutyl-1-methylxanthine (IBMX; 100 μM; A0695; AppliChem, Darmstadt, Germany), a non-specific inhibitor of the classical cardiac PDEs (IBMX *vs.* PF + IBMX: *p* = 0.670 in Ctl, *p* = 0.713 in AF).

**Fig. 1:**
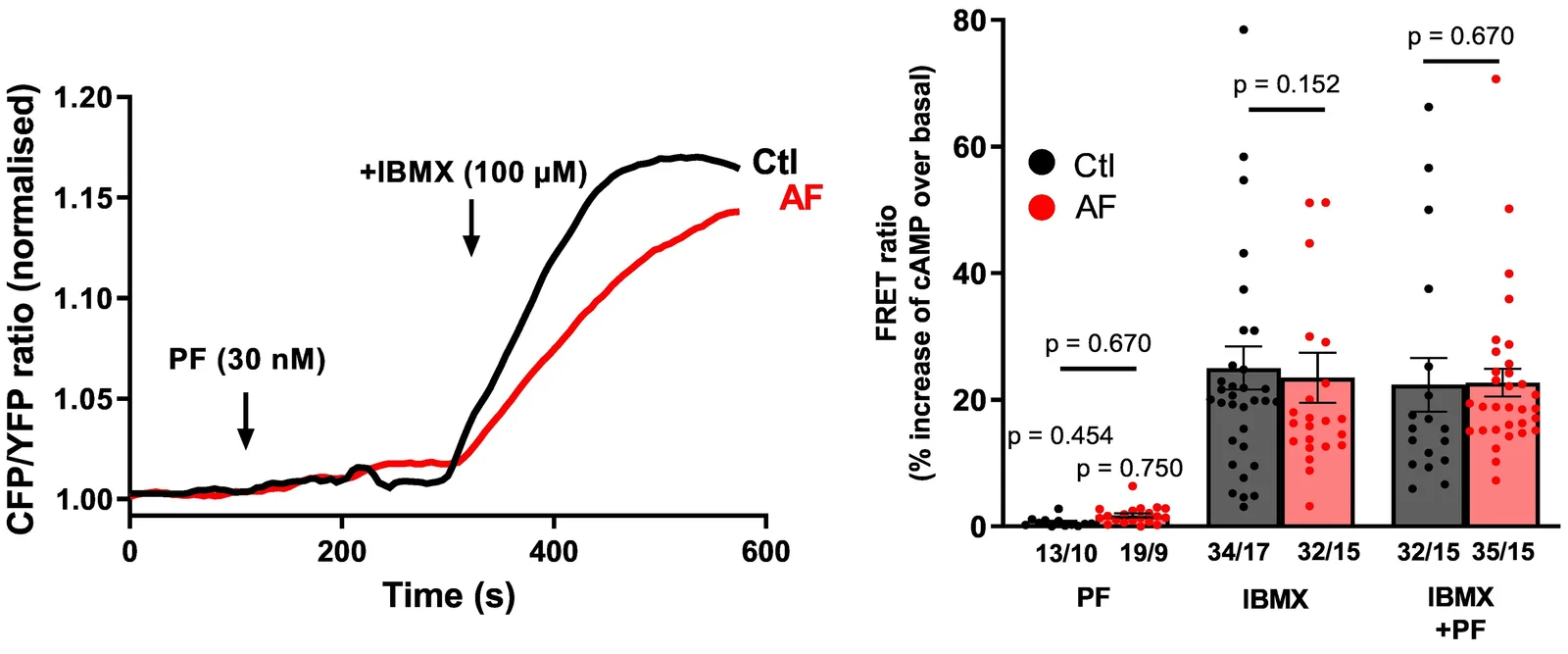
**PDE8 inhibition has minimal impact on global cAMP levels.** Left panel shows two overlayed representative traces of the fluctuations on the FRET ratio upon treatment with PF and IBMX in Ctl and AF cardiomyocytes. Representative FRET traces were smoothed using a Savitzky-Golay filter for display purposes. Right panel shows the percentual increase on FRET ratio following the treatment with PF, IBMX or combination of both. Dots represent individual measurements. Biological replicates (N) represent independent primary tissue isolations from distinct patients; technical replicates (n) represent individual single-cell recordings. Experiments were independently replicated across *N* = 22 patients (*n* = 67 cells) for the Ctl group and *N* = 20 patients (*n* = 69 cells) for the AF group. Bars represent the mean of the values and error bars the standard error of the mean (SEM). Numbers below the graph represent n/N = cells/patients. *P*-values are calculated by estimated marginal means (EMM) on linear mixed model (LMM), adjusted for relevant clinical covariates and corrected for False Discovery Rate (FDR) using the Benjamini-Hochberg procedure.

Due to the compartmentalized nature of cAMP signaling, the lack of effect of PDE8 on global levels of cAMP was not enough to rule out the potential proarrhythmic effects of PDE8 inhibition. Thus, we measured cAMP in relevant nanodomains for arrhythmogenesis in human atrial myocytes, namely RyR2, TpnI and MyBP-C.

### 3.3 PDE8 inhibition does not affect spontaneous Ca^2+^ release through the RyR2

As PDE8A was previously related to proarrhythmic effects through RyR2 interaction in rodents [19], we initially performed PDE8A and RyR2 Co-IP in both Ctl and AF atrial samples. PDE8A and RyR2 co-immunoprecipitated in both groups (Fig. 2A), confirming the association of both proteins within the same macromolecular complex in the human atria. Densitometry analysis showed no significant differences in the PDE8A/RyR2 ratio between Ctl and AF (1.00 ± 0.18 in Ctl *vs.* 1.15 ± 0.21 in AF, *p* = 0.566).

**Fig. 2:**
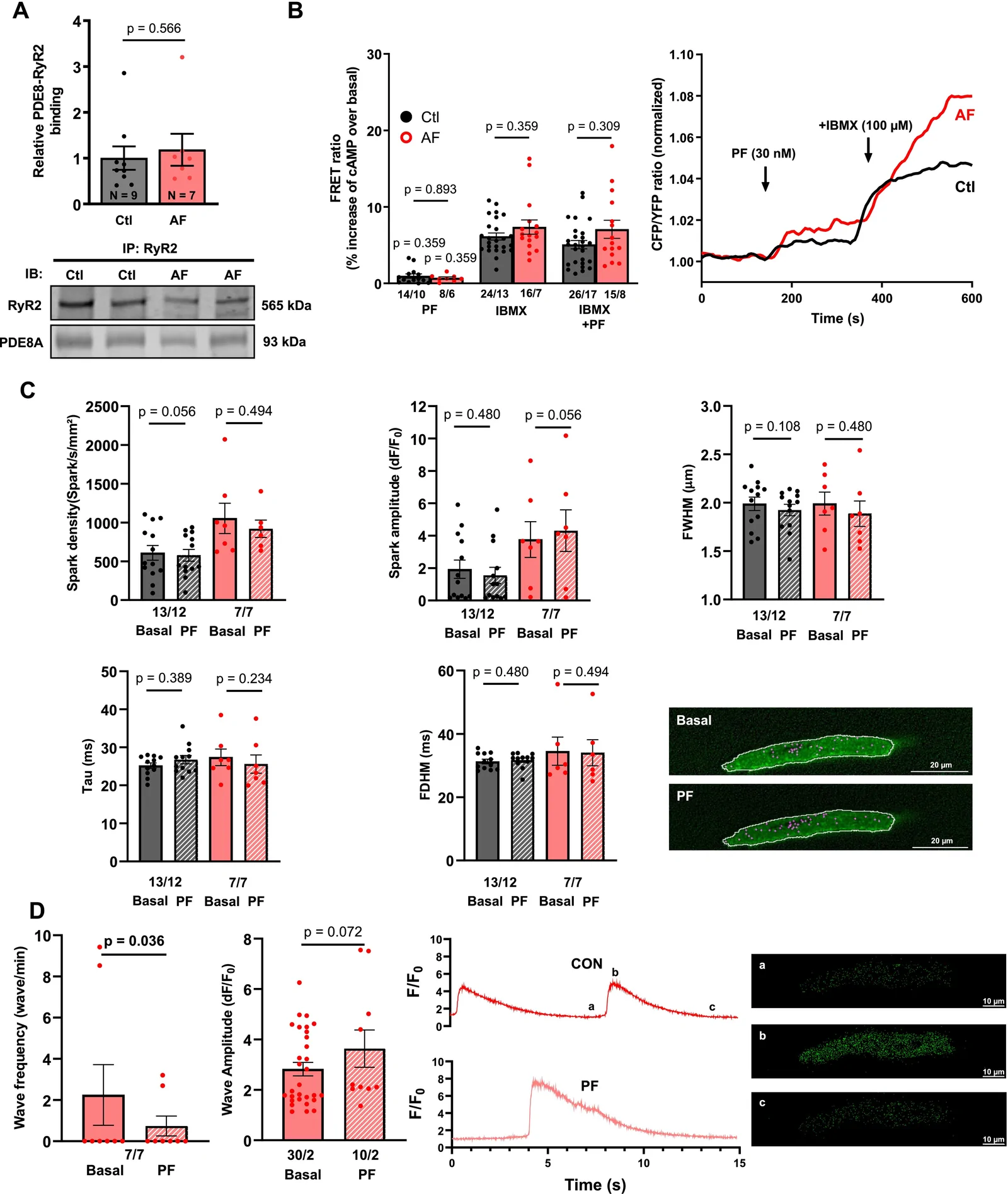
**PDE8 inhibition does not regulate RyR2 activity.** Representative blots of RyR2/PDE8A Co-IP and measured ratios of both proteins normalized to Ctl. Biological replicates (N) correspond to independent tissue homogenates from distinct patients (*N* = 9 for Ctl, *N* = 7 for AF. (B). Effects of PF and IBMX on RyR2 microdomain cAMP: left panel shows representative FRET ratio traces (smoothed with a Savitzky-Golay filter for display) and right panel shows cAMP increase upon PF, IBMX, and combined treatment. Biological replicates (N) represent independent patient tissue isolations; technical replicates (n) represent single-cell recordings (*N* = 13 patients, *n* = 54 cells for Ctl; *N* = 9 patients, *n* = 31 cells for AF). (C) Ca^2+^ spark density, amplitude, FWHM, tau, and FDHM in Ctl (*N* = 12 patients, *n* = 13 cells) and AF (*N* = 7 patients, *n* = 7 cells) cardiomyocytes before and after PF treatment. Lower right inset shows representative accumulated detected sparks in a Ctl cardiomyocyte at basal conditions and upon PF exposure. (D) Ca^2+^ wave frequency and amplitude in AF cardiomyocytes (*N* = 7 patients, *n* = 7 cells) before and after PF treatment, with representative wave traces and fluorescence intensity frames (a, b, c). Individual measurements are shown as dots. Bars represent group means and error bars represent SEM. Single-cell data were analyzed using Linear Mixed Models (LMM), except for wave frequency, which was analyzed using a Negative Binomial Generalized Linear Mixed Model (GLMM) with an offset for recording duration. Co-IP data were analyzed using an unpaired Student's *t*-test. All single-cell model *p*-values were adjusted for clinical covariates and FDR-corrected (Benjamini-Hochberg).

Next, we evaluated the effects of PF on cAMP levels in the RyR2 nanodomain. As observed in Fig. 2B, PF did not induce a significant increase in cAMP in this compartment (0.96 ± 0.28% in Ctl *vs.* 0.69 ± 0.16% in AF, *p* = 0.893; test against baseline: *p* = 0.359 in Ctl, *p* = 0.359 in AF). Moreover, PDE8 inhibition with PF did not increase the levels of cAMP beyond the IBMX-triggered response (*p* = 0.670 in Ctl, *p* = 0.713 in AF).

Then we studied the functional effects of PDE8 inhibition on spontaneous Ca^2+^ release from the sarcoplasmic reticulum through RyR2. We measured Ca^2+^ sparks and Ca^2+^ waves before and after PF application by confocal Ca^2+^ imaging. Characterization of spark properties showed that PDE8 inhibition does not result in significant changes in spark density, decay constant, FWHM, or FDHM in any of the groups after FDR correction (Fig. 2C, Supplementary Table 1). For spark amplitude in AF myocytes, PF treatment elicited an estimated increase (+0.45 dF/F0 , 95% CI [0.10,0.80]); however, this variation lost statistical significance after FDR adjustment (p  = 0.234, Supplementary Table 1). Regarding macroscopic events, PDE8 inhibition induced a statistically significant absolute reduction in Ca^2+^ wave frequency in AF myocytes (Estimate: −1.10 waves/min, 95% CI [−1.81,−0.38], *p* = 0.036, Fig. 2D). While macroscopic wave amplitude displayed broader variance in AF myocytes (Estimate: +0.98 dF/F0 , 95% CI [0.14,1.82]), this change was not statistically significant following FDR adjustment (*p* = 0.072, Supplementary Table 1). We did not detect any Ca^2+^ wave before or after the treatment in Ctl cells.

### 3.4 PDE8 does not regulate cAMP levels in sarcomeric compartments

To assess the role of PDE8 in sarcomeric cAMP regulation and contraction, we measured cAMP responses to PF in Ctl and AF myocytes expressing TpnI or MyBP-C targeted FRET biosensors. In the TpnI nanodomain, PDE8 inhibition did not elicit a significant change from the baseline (*p* = 0.859 in Ctl; *p* = 0.921 in AF) and these marginal changes were comparable between both groups (1.32 ± 0.23% in Ctl *vs.* 0.79 ± 0.14% in AF, *p* = 0.921; Fig. 3A). Similar results were obtained in the MyBP-C nanodomain, where the PF-induced cAMP increase was also negligible (*p* = 0.160 in Ctl; *p* = 0.174 in AF) and between groups (0.81 ± 0.17% in Ctl *vs.* 0.70 ± 0.12% in AF, *p* = 0.873; Fig. 3B). In contrast, the application of IBMX revealed a noticeable compartment-specific difference between groups. IBMX-induced cAMP increase was smaller in AF than in Ctl in the TpnI nanodomain, although this reduction did not reach statistical significance (8.94 ± 1.54% in Ctl *vs.* 5.08 ± 0.91% in AF, *p* = 0.270; Fig. 3A). Conversely, the response to IBMX in the MyBPC compartment was larger in AF compared to Ctl cardiomyocytes (2.65 ± 0.32% in Ctl *vs.* 4.45 ± 0.72% in AF, *p* = 0.024; Fig. 3B). Furthermore, PF failed to increase the response to IBMX (IBMX *vs.* PF + IBMX for TpnI: *p* = 0.921 in Ctl, *p* = 0.991 in AF; for MyBP-C: *p* = 0.813 in Ctl, *p* = 0.813 in AF).

**Fig. 3:**
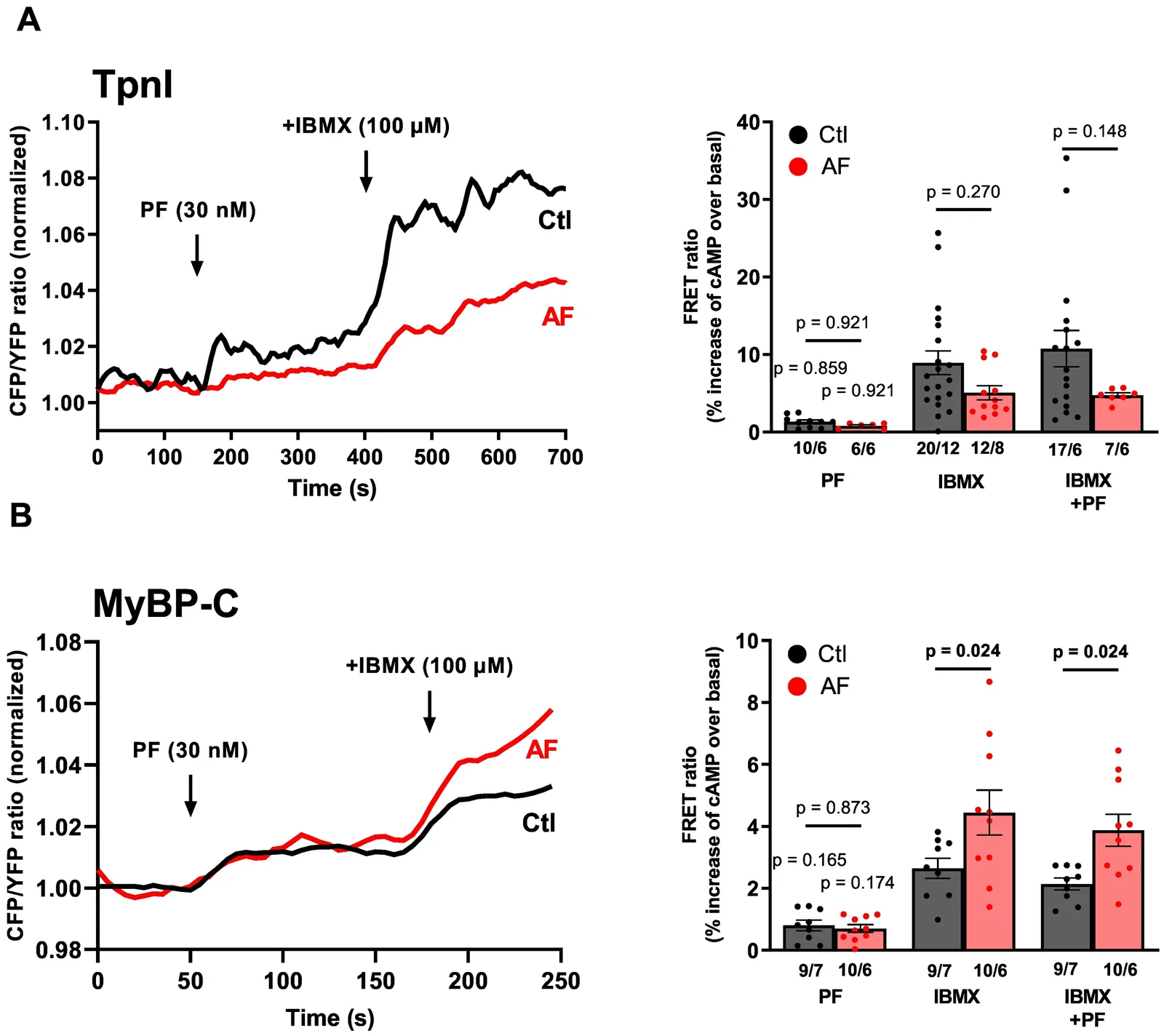
**PDE8 does not regulate cAMP levels in the sarcomeric nanodomains.** Real-time cAMP fluctuations in TpnI (A) and MyBP-C (B) nanodomains. Left panels show overlayed representative FRET ratio traces upon treatment with PF and IBMX in Ctl and AF cardiomyocytes (smoothed using a Savitzky-Golay filter for display purposes). Right panels show the percentage increase in FRET ratio following treatment with PF, IBMX, or their combination. Dots represent individual technical replicates (n, single-cell recordings) obtained from independent biological replicates (N, tissue isolations from distinct patients). For TpnI (A), experiments were replicated across *N* = 14 patients (*n* = 40 cells) for Ctl and *N* = 11 patients (*n* = 20 cells) for AF. For MyBP-C (B), experiments were replicated across *N* = 9 patients (*n* = 18 cells) for Ctl and *N* = 8 patients (*n* = 20 cells) for AF. Bars represent group means and error bars represent SEM. *P*-values were calculated from estimated marginal means (EMM) using Linear Mixed Models (LMM) with cells nested within patients as random effects, adjusted for clinical covariates and corrected for False Discovery Rate (FDR) using the Benjamini-Hochberg procedure.

## 4 Discussion

For decades, one of the main unsolved questions regarding AF pathophysiology was the mechanism underlying the reduction of I_Ca,L_ [15]. Our group demonstrated that this alteration is mediated by an increase in PDE8B expression and relocation to the L-type Ca^2+^ macromolecular complex in atrial myocytes from patients with persistent AF [18]. These results suggested that PDE8 inhibition could be used to restore I_Ca,L_ density in patients with AF. Here we studied the effects of PDE8 inhibition on other cAMP nanodomains. We used FRET to visualize cAMP levels in the cytosol, and in RyR2, TpnI and MyBP-C nanodomains, combined with confocal Ca^2+^ imaging to study spontaneous sarcoplasmic reticulum Ca^2+^ release. Our findings demonstrate that this pharmacological approach does not affect other compartments that could lead to arrhythmias in human atrial myocytes.

PDE8 is a cAMP-specific PDE with a very high affinity for cAMP [26]. This great affinity permits a fine regulation of cAMP signaling even with relatively low amounts of the enzyme [27], and when the enzyme is anchored to a precise subcellular location, compartmentation of cAMP can be extremely precise. Consistent with this concept, our results show that PDE8 inhibition has a negligible effect on cytosolic cAMP levels (Fig. 1). However, it was also important to ensure that total PDE8 inhibition (PDE8A and PDE8B inhibition) has no effect on other cAMP specific nanodomains, especially those governing the contractile machinery and sarcoplasmic reticulum Ca^2+^ release. Thus, the RyR2 nanodomain is of particular interest, as its PKA-dependent hyperphosphorylation is a known arrhythmogenic mechanism in AF [15], [16] and other types of arrhythmia [28].

We found that PDE8A co-precipitates with RyR2, but the PDE8A/RyR2 association ratio remained unaltered between Ctl and AF patients (Fig. 2A, Supplementary Table 1). This suggests that PDE8 signaling at the RyR2 nanodomain does not undergo pathophysiological remodeling in AF. Consistently, total PDE8 inhibition did not significantly alter cAMP levels in the RyR2 nanodomain (Fig. 2B), nor did it significantly change Ca^2+^ spark density, spatial spread (FWHM), or duration kinetics (FDHM and Tau) after False Discovery Rate (FDR) correction (Fig. 2C, Supplementary Table 1). For spark amplitude in AF myocytes, the minor increase observed in raw testing lost statistical significance following FDR adjustment (*p* = 0.234, Supplementary Table 1). Crucially, regarding macroscopic events, PDE8 inhibition did not merely fail to increase Ca^2+^ wave frequency, but induced a statistically significant absolute reduction in wave incidence in AF myocytes (Estimate: −1.10 waves/min, 95% CI [−1.81,−0.38], *p* = 0.036, Fig. 2D), confirming a functional protective effect. Although this reduction in wave frequency was accompanied by an estimated increase in wave amplitude (Estimate: +34.7%, 95% CI [0.14,1.82]) showing a noticeable effect size, this change lost statistical significance after FDR adjustment (*p* = 0.072, Supplementary Table 1).

Our results on Ca^2+^ imaging, however, contrast with previous observations in mouse ventricular myocytes, where PDE8A knock-out resulted in increased Ca^2+^ spark frequency and that suggested that PDE8A regulates the activity of RyR2 [19], raising concerns regarding potential pro-arrhythmic effects of PDE8 inhibition. Our data reveal that in human atrial myocytes, PDE8-mediated signaling remains confined to the L-type Ca^2+^ channel. Quantitative equivalence safety analysis in human atrial myocytes showed that the effects of total PDE8 inhibition on Ca^2+^ handling do not exceed the arrhythmogenic threshold (Supplementary Table 3) [24], [29], [30], [31]. Detailed evaluation of parameters exhibiting broader variance further supports functional safety. Specifically, for spark amplitude in AF myocytes, the estimated mean change (+13.4%) lost statistical significance after FDR adjustment (*p* = 0.234) and remained far below the >40% pathological threshold required for acute RyR2 cluster co-activation associated with overt arrhythmogenesis [30]. Regarding macroscopic events, while wave amplitude displayed a noticeable effect size trend (+34.7%), this variation was not statistically significant following FDR adjustment (*p* = 0.072). Crucially, any potential arrhythmogenic risk from an increased wave amplitude is mitigated by a statistically significant absolute reduction in wave frequency (*p* = 0.036). This marked decrease minimizes global event incidence and prevents the spatiotemporal I_TI_ summation. Consistently, it prevents electrogenic NCX1 depolarizations exceeding the >15 mV threshold for DAD induction [24], [29], [32]. These discrepancies also highlight fundamental differences between rodent ventricular and human atrial architecture, as rodent ventricular cardiomyocytes exhibit a dense network of T-tubules, whereas human atrial cardiomyocytes lack of a highly structured T-tubules network [33], [34], [35], [36]. This difference in cell architecture profoundly affects Ca^2+^ dynamics [33], [34], [37]. Furthermore, it is well-established that the expression and compartmentation of PDEs significantly varies between species and chambers [11], [28], [38], [39], [40]. However, aiming for precision pharmacological interventions, it would be of major help to develop alternatives that specifically target the PDE8B isoform. This could be achieved either through the development of true isoform-selective inhibitors or by using novel approaches designed to specifically target and disrupt the association of PDE8B with the L-type Ca^2+^ channel complex.

To evaluate the effects of PDE8 inhibition on the contractile apparatus, we additionally measured the fluctuations in cAMP levels following PDE8 inhibition in the TpnI and MyBP-C nanodomains. Similar to what we observed for the RyR2, PDE8 inhibition did not significantly increase cAMP in these compartments (Fig. 3A-B). These results are in line with a recent study conducted in human trabeculae from patients in sinus rhythm or with persistent AF [41] that showed no effect of PDE8 inhibition on atrial tissues in neither force of contraction nor arrhythmogenesis, in basal or upon β-adrenergic stimulation, in either group of patients. Importantly, PDE8 inhibition did not increase the incidence of norepinephrine-induced arrhythmias in the human trabeculae [41]. The absence of cAMP accumulation at the myofilaments confirms that PDE8 inhibition avoids off-target perturbations at the sarcomere level. It is well known that altered myofilament phosphorylation changes Ca^2+^ sensitivity, which can modify cytosolic Ca^2+^ buffering dynamics and potentially promote arrhythmias [42], [43]. Therefore, the fact that PDE8 inhibition does not affect these nanodomains reinforces its high spatial selectivity and reduces concerns regarding potential alterations of myofilament mechanics.

Interestingly, while PDE8 seems to have no effect in the sarcomeric regulation of cAMP, the application of IBMX revealed a profound nanoscale-resolution remodeling of the classical cardiac PDEs in AF. We observed opposite behaviors in the cAMP response to IBMX in the adjacent compartments TpnI and MyBP-C. While in TpnI AF cardiomyocytes exhibited a blunted response to IBMX, in MyBP-C they displayed a massive response compared to Ctl cardiomyocytes. This provides direct functional evidence in human cells for the highly compartmentalized nature of cAMP signaling, supporting the previously proposed nanometer-scale compartmentation [44]. From a pathophysiological point of view, this opposite trend in cAMP tone could lead to PKA phosphorylation imbalance within the sarcomere. Specifically, this mismatch in nanodomains regulating lusotropy (TpnI) [45] and inotropy (MyBP-C) [46], could provide a molecular basis for contractile dysfunction, a common feature in AF [47], [48]. In any case, the remodeling of PDEs in the sarcomere does not include PDE8.

Altogether these results suggest that acute PDE8 inhibition does not promote spontaneous arrhythmogenic Ca^2+^ releases in isolated human atrial myocytes.

### 4.1 Limitations

Our study evaluates the acute effects of PDE8 inhibition on relevant cAMP nanodomains and spontaneous sarcoplasmic reticulum Ca^2+^ release in isolated human atrial myocytes. We did not detect pro-arrhythmic effects in the evaluated parameters. However, we did not evaluate action potentials or chronic exposure, limiting the translation of these findings to overall antiarrhythmic safety. Furthermore, there is an imbalance in several of the patients' clinical characteristics across groups, which arise from the intrinsic features of the surgical procedures. One of the most notorious is the ratio of left and right atrial samples. A big share of the samples included in the AF group came from the left atrium. Likewise, the AF cohort is inherently older and is more commonly prescribed beta-blockers and diuretics, which reflects the standard characteristics of the disease and first line treatment. Rather than discarding valuable samples to match the baseline characteristics of both cohorts, we implemented hierarchical LMM and GLMM with nested random effects (cells within patients) and empirical covariate screening (with a limit of one covariate per 10 patients to prevent model overfitting). Combined with False Discovery Rate (FDR) adjustment, this advanced statistical pipeline rigorously controlled for Type I error inflation and isolated experimental treatment effects from clinical confounding factors. Additionally, and although the membrane and structural integrity of the myocytes during culture was previously confirmed [49], we cannot completely rule out that alterations may occur due to the 24 to 48 h *in vitro* culture period required to perform FRET experiments.

## 5 Conclusion

Altogether, and in contrast with other PDEs that simultaneously regulate different cAMP nanodomains, the highly confined localization of PDE8B in the L-type Ca^2+^ channel provides a unique pharmacological profile. Due to this precise compartmentation, total PDE8 inhibition can be used to normalize I_Ca,L_ without acutely promoting arrhythmogenic sarcoplasmic reticulum Ca^2+^ releases in human atrial myocytes.

## CRediT authorship contribution statement

**David Revuelta:** Writing – original draft, Methodology, Investigation, Formal analysis. **Nefeli Grammatika Pavlidou:** Investigation. **Cristina Aceituno:** Investigation. **Issam Abu Taha:** Investigation. **Andrea Brunetti:** Investigation. **Antonino Ginel:** Resources. **Evaldas Girdauskas:** Resources. **Dobromir Dobrev:** Validation, Resources. **Leif Hove-Madsen:** Investigation, Funding acquisition. **Cristina E. Molina:** Investigation.

## Declaration of Generative AI and AI-assisted technologies in the writing process

During the preparation of this work, the authors used Gemini (version 3.1 Pro; Google LLC, Mountain View, CA, USA) to improve English grammar, conciseness, word choice, and overall readability. After using this tool, the authors carefully reviewed and edited the text as needed, and take full responsibility for the final content of the publication. The authors explicitly state that this AI tool was not used to generate original scientific content, interpret experimental results, draw conclusions, or analyze data, nor did it have access to any raw datasets at any point.

## Funding

This research was supported by the 10.13039/501100001659Deutsche Forschungsgemeinschaft (ES 569/2–2 and ES 569/3–1 to CM), the Gertraud and Heinz-Rose Foundation (to CM), the 10.13039/100010447German Centre for Cardiovascular Research (DZHK), and the Spanish Ministry of Science Innovation and Universities (PID2023–152610OB-C21 to LHM).

## Declaration of competing interest

The authors declare no conflict of interest.

## Data Availability

Data are available upon request.

## Appendix A Supplementary data

Supplementary figures

Supplementary table 1 **Statistical summary of experiments.** Quantitative evaluation of live-cell FRET ratio changes, Ca^2+^ spark and wave parameters, and RyR2–PDE8A co-immunoprecipitation in control (Ctl) and atrial fibrillation (AF) myocytes upon total PDE8 inhibition with PF. Estimates, 95% CIs, and Cohen's *d* effect sizes were derived from Linear Mixed Models (LMM) with cells nested within patients as random effects, adjusted for clinical covariates. Ca^2+^ wave frequency was analyzed using a negative binomial Generalized Linear Mixed Model (GLMM) with an offset term. All *p*-values were adjusted for multiple testing using False Discovery Rate (FDR) correction (Benjamini–Hochberg). Abbreviations: AF, atrial fibrillation; CI, confidence interval; Co-IP, co-immunoprecipitation; Ctl, control; FDR, false discovery rate; FRET, fluorescence resonance energy transfer; GLMM, generalized linear mixed model; LMM, linear mixed model; PDE8, phosphodiesterase 8; RyR2, ryanodine receptor 2.

Supplementary table 2 **Sample size allocation, clinical profiles, and tissue fate breakdown across experimental assays.** (A) Summary of cell (n) and patient (N) sample sizes contributing to each experimental sub-assay (FRET live-cell imaging, confocal Ca^2+^ imaging, and Co-IP) in control (Ctl) and atrial fibrillation (AF) cohorts. (B) Individual patient breakdown detailing clinical group, atrial chamber origin (right *vs.* left atrium), AF classification, surgical intervention type, prior pharmacological exposure, and specific experimental assay allocation. Abbreviations: AA, antiarrhythmic drugs; AB, angiotensin II receptor blockers; AF, atrial fibrillation; AI, angiotensin converting enzyme inhibitors; BB, beta-blockers; C, coronary artery bypass surgery; CA, Ca^2+^ channel antagonists; Co-IP, co-immunoprecipitation; Ctl, control; D, diuretics; FRET, fluorescence resonance energy transfer; LA, left atrium; N, patient count; n, cell count; N/A, not applicable; N, nitrates; O (Intervention), other surgical procedures; O (Treatment), other pharmacological treatments; RA, right atrium; SR, sinus rhythm; ST, statins; V, valve surgery.

Supplementary table 3 **Objective equivalence safety analysis of total PDE8 inhibition in AF myocytes.** Functional equivalence evaluation of selective PDE8 inhibition (30 nM PF) on spontaneous Ca^2+^ release dynamics in human AF atrial myocytes. Point estimates and upper 95% CIs were compared against pre-specified objective safety margins (Δ) derived using the half-pathological shift (Δ = Minimum Documented Pathological Shift/2). *P*-values were derived from LMM or GLMM models with FDR adjustment. AF, atrial fibrillation; CI, confidence interval; FDR, false discovery rate; GLMM, generalized linear mixed model; LMM, linear mixed model; PDE8, phosphodiesterase 8; RyR2, ryanodine receptor 2.

## References

[1] Bers D.M. (2002). Cardiac excitation-contraction coupling. Nature.

[2] Tamborrini D., Wang Z., Wagner T., Tacke S., Stabrin M., Grange M. (2023). Structure of the native myosin filament in the relaxed cardiac sarcomere. Nature.

[3] Jurevicius J., Fischmeister R. (1996). cAMP compartmentation is responsible for a local activation of cardiac Ca2+ channels by beta-adrenergic agonists. Proc Natl Acad Sci USA.

[4] Marx S.O., Reiken S., Hisamatsu Y., Jayaraman T., Burkhoff D., Rosemblit N. (2000). PKA phosphorylation dissociates FKBP12.6 from the calcium release channel (ryanodine receptor): defective regulation in failing hearts. Cell.

[5] Dodge-Kafka K.L., Soughayer J., Pare G.C., Carlisle Michel J.J., Langeberg L.K., Kapiloff M.S. (2005). The protein kinase a anchoring protein mAKAP coordinates two integrated cAMP effector pathways. Nature.

[6] Folkmanaite M., Zaccolo M. (2023). Regulation of cardiac function by cAMP nanodomains. Biosci Rep.

[7] Zaccolo M., Pozzan T. (2002). Discrete microdomains with high concentration of cAMP in stimulated rat neonatal cardiac myocytes. Science.

[8] Sadayappan S., Gulick J., Osinska H., Martin L.A., Hahn H.S., Dorn G.W. (2005). Cardiac myosin-binding protein-C phosphorylation and cardiac function. Circ Res.

[9] Mika D., Bobin P., Pomérance M., Lechêne P., Westenbroek R.E., Catterall W.A. (2013). Differential regulation of cardiac excitation-contraction coupling by cAMP phosphodiesterase subtypes. Cardiovasc Res.

[10] Chao Y.C., McAndrew D.J., Revuelta D., Yadav R., Hong W.H., Hu J. (2025). Targeting Troponin I Phosphorylation for Precision Treatment of Diastolic Dysfunction. bioRxiv.

[11] Molina C.E., Leroy J., Richter W., Xie M., Scheitrum C., Lee I.-O. (2012). Cyclic adenosine monophosphate phosphodiesterase type 4 protects against atrial arrhythmias. J Am Coll Cardiol.

[12] Dolce B., Christ T., Grammatika Pavlidou N., Yildirim Y., Reichenspurner H., Eschenhagen T. (2021). Impact of phosphodiesterases PDE3 and PDE4 on 5-hydroxytryptamine receptor4-mediated increase of cAMP in human atrial fibrillation. Naunyn Schmiedeberg’s Arch Pharmacol.

[13] Garnier A., Bork N.I., Jacquet E., Zipfel S., Muñoz-Guijosa C., Baczkó I. (2021). Mapping genetic changes in the cAMP-signaling cascade in human atria. J Mol Cell Cardiol.

[14] Dorey T.W., Liu Y., Jansen H.J., Bohne L.J., Mackasey M., Atkinson L. (2023). Natriuretic peptide receptor B protects against atrial fibrillation by controlling atrial cAMP via phosphodiesterase 2. Circ Arrhythm Electrophysiol.

[15] Reinhardt F., Beneke K., Pavlidou N.G., Conradi L., Reichenspurner H., Hove-Madsen L. (2021). Abnormal calcium handling in atrial fibrillation is linked to changes in cyclic AMP dependent signaling. Cells.

[16] Vest J.A., Wehrens X.H.T., Reiken S.R., Lehnart S.E., Dobrev D., Chandra P. (2005). Defective cardiac ryanodine receptor regulation during atrial fibrillation. Circulation.

[17] El-Armouche A., Boknik P., Eschenhagen T., Carrier L., Knaut M., Ravens U. (2006). Molecular determinants of altered Ca2+ handling in human chronic atrial fibrillation. Circulation.

[18] Grammatika Pavlidou N., Dobrev S., Beneke K., Reinhardt F., Pecha S., Jacquet E. (2023). Phosphodiesterase 8 governs cAMP/PKA-dependent reduction of L-type calcium current in human atrial fibrillation: a novel arrhythmogenic mechanism. Eur Heart J.

[19] Patrucco E., Albergine M.S., Santana L.F., Beavo J.A. (2010). Phosphodiesterase 8A (PDE8A) regulates excitation-contraction coupling in ventricular myocytes. J Mol Cell Cardiol.

[20] Mika D., Leroy J., Vandecasteele G., Fischmeister R. (2012). PDEs create local domains of cAMP signaling. J Mol Cell Cardiol.

[21] Nikolaev V.O., Bünemann M., Hein L., Hannawacker A., Lohse M.J. (2004). Novel single chain cAMP sensors for receptor-induced signal propagation. J Biol Chem.

[22] Berisha F., Götz K.R., Wegener J.W., Brandenburg S., Subramanian H., Molina C.E. (2021). cAMP imaging at ryanodine receptors reveals β2-adrenoceptor driven arrhythmias. Circ Res.

[23] Surdo N.C., Berrera M., Koschinski A., Brescia M., Machado M.R., Carr C. (2017). FRET biosensor uncovers cAMP nano-domains at β-adrenergic targets that dictate precise tuning of cardiac contractility. Nat Commun.

[24] Tarifa C., Vallmitjana A., Jiménez-Sábado V., Marchena M., Llach A., Herraiz-Martínez A. (2023). Spatial distribution of calcium Sparks determines their ability to induce afterdepolarizations in human atrial myocytes. JACC Basic Transl Sci.

[25] Benjamini Y., Hochberg Y. (1995). Controlling the false discovery rate: a practical and powerful approach to multiple testing. J R Stat Soc Ser B Methodol.

[26] Soderling S.H., Bayuga S.J., Beavo J.A. (1998). Cloning and characterization of a cAMP-specific cyclic nucleotide phosphodiesterase. Proc Natl Acad Sci USA.

[27] Brown K.M., Day J.P., Huston E., Zimmermann B., Hampel K., Christian F. (2013). Phosphodiesterase-8A binds to and regulates Raf-1 kinase. Proc Natl Acad Sci USA.

[28] Lehnart S.E., Wehrens X.H.T., Reiken S., Warrier S., Belevych A.E., Harvey R.D. (2005). Phosphodiesterase 4D deficiency in the ryanodine-receptor complex promotes heart failure and arrhythmias. Cell.

[29] Hove-Madsen L., Llach A., Bayes-Genís A., Roura S., Font E.R., Arís A. (2004). Atrial fibrillation is associated with increased spontaneous calcium release from the sarcoplasmic reticulum in human atrial myocytes. Circulation.

[30] Nolla-Colomer C., Casabella-Ramon S., Jimenez-Sabado V., Vallmitjana A., Tarifa C., Herraiz-Martínez A. (2022). β2-adrenergic stimulation potentiates spontaneous calcium release by increasing signal mass and co-activation of ryanodine receptor clusters. Acta physiologica (Oxford, England).

[31] Casabella-Ramón S., Jiménez-Sábado V., Tarifa C., Casellas S., Lu T.T., Izquierdo-Castro P. (2022). Impact of R-carvedilol on β2-adrenergic receptor-mediated spontaneous calcium release in human atrial myocytes. Biomedicines.

[32] Schlotthauer K., Bers D.M. (2000). Sarcoplasmic reticulum ca(2+) release causes myocyte depolarization. Underlying mechanism and threshold for triggered action potentials. Circ Res.

[33] Wagner E., Brandenburg S.S., Kohl T.T., Lehnart S.E. (2014). Analysis of tubular membrane networks in cardiac myocytes from atria and ventricles. J Vis Exp.

[34] Brandenburg S., Kohl T., Williams G.S., Gusev K., Wagner E., Rog-Zielinska E.A. (2016). Axial tubule junctions control rapid calcium signaling in atria. J Clin Invest.

[35] Greiser M. (2017). Calcium signalling silencing in atrial fibrillation. J Physiol.

[36] Vagos M.R., Arevalo H., Heijman J., Schotten U., Sundnes J. (2020). A novel computational model of the rabbit atrial cardiomyocyte with spatial calcium dynamics. Front Physiol.

[37] Walden A.P., Dibb K.M., Trafford A.W. (2009). Differences in intracellular calcium homeostasis between atrial and ventricular myocytes. J Mol Cell Cardiol.

[38] Leroy J., Richter W., Mika D., Castro L.R.V., Abi-Gerges A., Xie M. (2011). Phosphodiesterase 4B in the cardiac L-type Ca2+ channel complex regulates Ca2+ current and protects against ventricular arrhythmias in mice. J Clin Invest.

[39] Kamel R., Leroy J., Vandecasteele G., Fischmeister R. (2023). Cyclic nucleotide phosphodiesterases as therapeutic targets in cardiac hypertrophy and heart failure. Nat Rev Cardiol.

[40] Molenaar P., Christ T., Hussain R.I., Engel A., Berk E., Gillette K.T. (2013). PDE3, but not PDE4, reduces β1- and β2-adrenoceptor-mediated inotropic and lusitropic effects in failing ventricle from metoprolol-treated patients. Br J Pharmacol.

[41] Ismaili D., Petersen J., Schulz C., Eschenhagen T., Koivumäki J.T., Christ T. (2024). PDE8 inhibition and its impact on ICa,L in persistent atrial fibrillation: evaluation of PDE8 as a potential drug target. J Cardiovasc Pharmacol.

[42] Baudenbacher F., Schober T., Pinto J.R., Sidorov V.Y., Hilliard F., Solaro R.J. (2008). Myofilament Ca2+ sensitization causes susceptibility to cardiac arrhythmia in mice. J Clin Invest.

[43] Santini L., Coppini R., Cerbai E. (2021). Ion Channel impairment and myofilament Ca2+ sensitization: two parallel mechanisms underlying Arrhythmogenesis in hypertrophic cardiomyopathy. Cells.

[44] Chao Y.-C., Surdo N.C., Pantano S., Zaccolo M. (2019). Imaging cAMP nanodomains in the heart. Biochem Soc Trans.

[45] Messer A.E., Marston S.B. (2014). Investigating the role of uncoupling of troponin I phosphorylation from changes in myofibrillar Ca2+−sensitivity in the pathogenesis of cardiomyopathy. Front Physiol.

[46] Tong C.W., Wu X., Liu Y., Rosas P.C., Sadayappan S., Hudmon A. (2015). Phosphoregulation of cardiac Inotropy via myosin binding protein-C during increased pacing frequency or β1-adrenergic stimulation. Circ Heart Fail.

[47] Goette A., Kalman J.M., Aguinaga L., Akar J., Cabrera J.A., Chen S.A. (2016). EHRA/HRS/APHRS/SOLAECE expert consensus on atrial cardiomyopathies: definition, characterisation, and clinical implication. J Arrhythm.

[48] Van Gelder I.C., Rienstra M., Bunting K.V., Casado-Arroyo R., Caso V., Crijns H.J.G.M. (2024). 2024 ESC guidelines for the management of atrial fibrillation developed in collaboration with the European Association for Cardio-Thoracic Surgery (EACTS). Eur Heart J.

[49] Beneke K., Molina C.E. (2022). Live cell imaging of cyclic nucleotides in human cardiomyocytes. Methods Mol Biol (Clifton, NJ).

