# Supplementary material for: Absence of arrhythmogenic effects in cAMP subcellular compartments supports PDE8 as a target in atrial fibrillation
Source: J Mol Cell Cardiol Plus. 2026 Sep 26;18:100871. doi: 10.1016/j.jmccpl.2026.100871 (PMC13673357; doi:10.1016/j.jmccpl.2026.100871)
Supplement: Supplementary file 1 [file mmc1.pdf]

RAW

Gel 1

IP: RyR2

IB:

RyR2

565 kDa

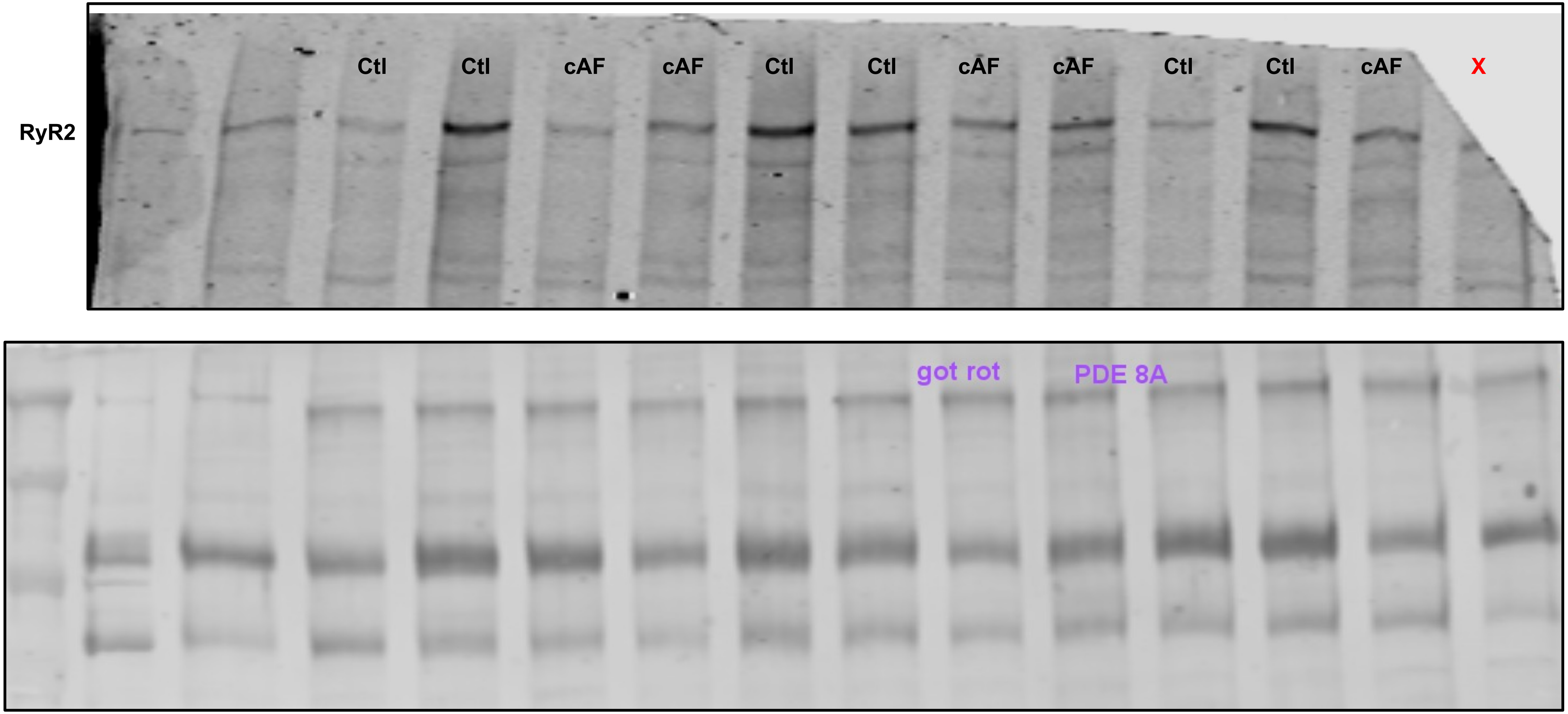

PDE8A

100 kDa

75 kDa

Scaned with Image Studio
at a Resolution of 169µm

RAW

Gel 2

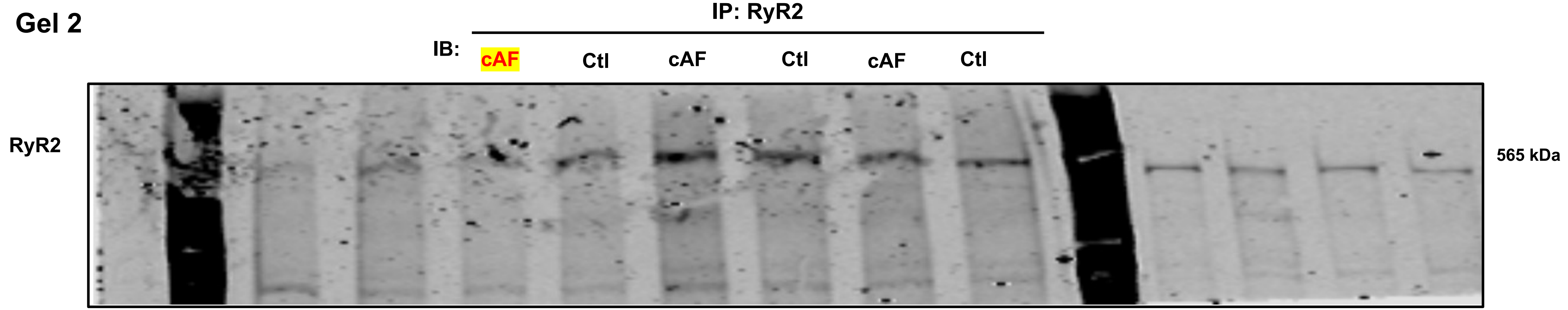

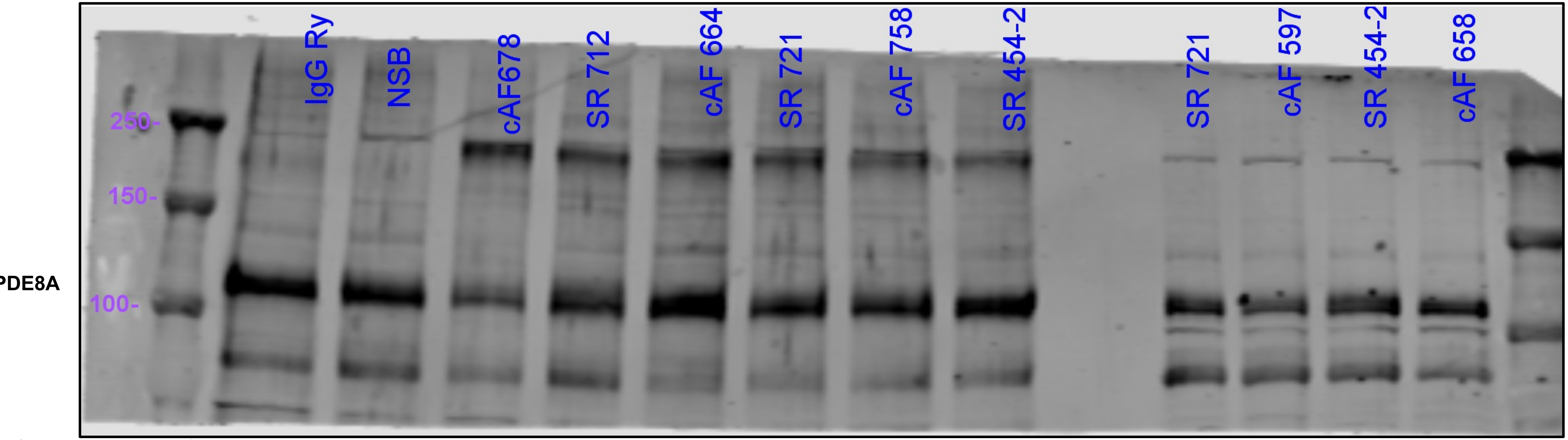

Scaned with Image Studio
at a Resolution of 169µm
