# Supplementary material for: Absence of arrhythmogenic effects in cAMP subcellular compartments supports PDE8 as a target in atrial fibrillation
Source: J Mol Cell Cardiol Plus. 2026 Sep 26;18:100871. doi: 10.1016/j.jmccpl.2026.100871 (PMC13673357; doi:10.1016/j.jmccpl.2026.100871)
Supplement: Supplementary file 2 [file mmc2.docx]

| **FRET** | | | | | | | |
| --- | --- | --- | --- | --- | --- | --- | --- |
| **Parameter (Group)** | **Comparison** | **Estimate (Change)** | **95% CI of Estimate** | **Effect Size (Cohen's d)** | **95% CI of Effect Size** | **p-value** | **Adjusted p-value** |
| **Cytosolic cAMP (SR)** | PF vs. Baseline | +5.48 % | [-2.98, 13.95] | 0.27 | [-0.14, 0.67] | 0.202 | 0.454 |
|  | IBMX vs. Baseline | +28.84 % | [21.84, 35.83] | 2.25 | [1.57, 2.93] | <0.001 | **<0.001** |
|  | IBMX vs. PF+IBMX | +3.17 % | [-3.71, 10.05] | *N/A* | *N/A* | 0.521 | 0.670 |
| **Cytosolic cAMP (AF)** | PF vs. Baseline | +1.29 % | [-6.77, 9.35] | 0.08 | [-0.39, 0.54] | 0.750 | 0.750 |
|  | IBMX vs. Baseline | +18.76 % | [11.47, 26.05] | 1.41 | [0.80, 2.00] | <0.001 | **<0.001** |
|  | IBMX vs. PF+IBMX | -2.54 % | [-9.13, 4.06] | *N/A* | *N/A* | 0.634 | 0.713 |
| **Cytosolic cAMP (SR vs. AF)** | PF | +4.19% | [-7.50, 15.88] | *N/A* | *N/A* | 0.478 | 0.670 |
|  | IBMX | +10.08% | [-0.03, 20.18] | *N/A* | *N/A* | 0.051 | 0.152 |
|  | IBMX+PF | +4.37% | [-5.93, 14, 67 | *N/A* | *N/A* | 0.399 | 0.670 |
| **RyR2 cAMP (SR)** | PF vs. Baseline | +1.20 % | [-0.66, 3.07] | 0.41 | [-0.21, 1.02] | 0.200 | 0.359 |
|  | IBMX vs. Baseline | +6.48 % | [4.82, 8.14] | 2.96 | [1.90, 4.00] | <0.001 | **<0.001** |
|  | IBMX vs. PF+IBMX | +1.11 % | [-0.52, 2.73] | *N/A* | *N/A* | 0.240 | 0.359 |
| **RyR2 cAMP (AF)** | PF vs. Baseline | +1.60 % | [-0.73, 3.92] | 0.40 | [-0.18, 0.97] | 0.175 | 0.359 |
|  | IBMX vs. Baseline | +7.89 % | [5.86, 9.92] | 2.90 | [1.87, 3.91] | <0.001 | **<0.001** |
|  | IBMX vs. PF+IBMX | +0.33 % | [-1.78, 2.45] | *N/A* | *N/A* | 0.925 | 0.925 |
| **RyR2 cAMP (SR vs. AF)** | PF | -0.39 % | [-3.38, 2.60] | *N/A* | *N/A* | 0.794 | 0.893 |
|  | IBMX | -1.42 % | [-4.04, 1.21] | *N/A* | *N/A* | 0.279 | 0.359 |
|  | IBMX+PF | -2.19 % | [-4.85, 0.47] | *N/A* | *N/A* | 0.103 | 0.309 |
| **MyBP-C cAMP (SR)** | PF vs. Baseline | +0.81 % | [-0.13, 1.74] | 0.58 | [-0.09, 1.24] | 0.089 | 0.160 |
|  | IBMX vs. Baseline | +2.65 % | [1.66, 3.63] | 2.13 | [1.17, 3.06] | <0.001 | **<0.001** |
|  | IBMX vs. PF+IBMX | +0.50 % | [-1.08, 2.09] | *N/A* | *N/A* | 0.722 | 0.813 |
| **MyBP-C cAMP (AF)** | PF vs. Baseline | +0.70 % | [-0.18, 1.59] | 0.56 | [-0.14, 1.25] | 0.116 | 0.174 |
|  | IBMX vs. Baseline | +4.45 % | [3.53, 5.36] | 4.04 | [2.65, 5.40] | <0.001 | **<0.001** |
|  | IBMX vs. PF+IBMX | +0.57 % | [-0.95, 2.09] | *N/A* | *N/A* | 0.637 | 0.813 |
| **MyBP-C cAMP (SR vs. AF)** | PF | +0.10 % | [-1.19, 1.39] | *N/A* | *N/A* | 0.873 | 0.873 |
|  | IBMX | -1.80 % | [-3.15, 0.45] | *N/A* | *N/A* | 0.011 | **0.024** |
|  | IBMX+PF | -1.73 % | [-3.02, 0.44] | *N/A* | *N/A* | 0.010 | **0.024** |
| **TpnI cAMP (SR)** | PF vs. Baseline | +1.51 % | [-2.74, 5.76] | 0.22 | [-0.38, 0.81] | 0.477 | 0.859 |
|  | IBMX vs. Baseline | +8.94 % | [6.02, 11.87] | 1.98 | [1.21, 2.74] | <0.001 | **<0.001** |
|  | IBMX vs. PF+IBMX | -1.63 % | [-6.72, 3.47] | *N/A* | *N/A* | 0.726 | 0.921 |
| **TpnI cAMP (AF)** | PF vs. Baseline | +0.74 % | [-4.48, 5.96] | 0.07 | [-0.44, 0.58] | 0.778 | 0.921 |
|  | IBMX vs. Baseline | +5.12 % | [1.22, 9.02] | 0.94 | [0.21, 1.66] | 0.012 | 0.052 |
|  | IBMX vs. PF+IBMX | +0.40 % | [-6.90, 7.69] | *N/A* | *N/A* | 0.991 | 0.991 |
| **TpnI cAMP (SR vs. AF)** | PF | + 0.77 % | [-5.96, 7.50] | *N/A* | *N/A* | 0.818 | 0.921 |
|  | IBMX | + 3.82 % | [-1.05, 8.69] | *N/A* | *N/A* | 0.120 | 0.270 |
|  | IBMX+PF | + 5.85 % | [0.02, 11.67] | *N/A* | *N/A* | 0.049 | 0.148 |
| **Ca^2+^ imaging** | | | | | | | |
| **Parameter (Group)** | **Comparison** | **Estimate (Change)** | **95% CI of Estimate** | **Effect Size (Cohen's d)** | **95% CI of Effect Size** | **p-value** | 0.494 |
| **Spark Density (SR), spark/s/mm^2^** | PF vs. Baseline | -78.69 | [-137.56, -19.82] | -1.11 | [-1.98, -0.23] | 0.012 | 0.056 |
| **Spark Density (AF), spark/s/mm^2^** | PF vs. Baseline | +28.69 | [-57.88, 115.25] | +0.40 | [-0.82, 1.62] | 0.494 | 0.494 |
| **Spark Amplitude (SR), dF/F_0_** | PF vs. Baseline | -0.11 | [-0.37, 0.15] | -0.35 | [-1.18, 0.48] | 0.385 | 0.480 |
| **Spark Amplitude (AF) ), dF/F_0_** | PF vs. Baseline | +0.45 | [0.10, 0.80] | +1.45 | [0.26, 2.63] | 0.014 | 0.056 |
| **Spark Tau (SR), ms** | PF vs. Baseline | +1.26 | [-0.86, 3.38] | +0.51 | [-0.35, 1.36] | 0.227 | 0.389 |
| **Spark Tau (AF), ms** | PF vs. Baseline | -2.18 | [-4.97, 0.61] | -0.88 | [-2.01, 0.25] | 0.117 | 0.234 |
| **Spark FWHM (SR), µm** | PF vs. Baseline | -0.09 | [-0.18, -0.00] | -0.84 | [-1.70, 0.01] | 0.045 | 0.108 |
| **Spark FWHM (AF), µm** | PF vs. Baseline | -0.05 | [-0.18, 0.07] | -0.46 | [-1.58, 0.66] | 0.400 | 0.480 |
| **Spark FDHM (SR), ms** | PF vs. Baseline | +0.71 | [-0.95, 2.37] | +0.38 | [-0.52, 1.29] | 0.399 | 0.480 |
| **Spark FDHM (AF), ms** | PF vs. Baseline | -0.84 | [-2.92, 1.25] | -0.45 | [-1.59, 0.68] | 0.478 | 0.494 |
| **Wave Frequency (AF), waves/min** | PF vs. Baseline | -1.10 | [-1.81, -0.38] | -1.50 | [-2.48, -0.52] | 0.003 | **0.036** |
| **Wave Amplitude (AF) dF/F_0_** | PF vs. Baseline | +0.98 | [0.14, 1.82] | +0.86 | [-3.82, 5.55] | 0.024 | 0.072 |
| **Co-IP** | | | | | | |  |
| **Parameter (Group)** | **Comparison** | **Estimate (Change)** | **95% CI of Estimate** | **Effect Size (Cohen's d)** | **95% CI of Effect Size** | **p-value** | **Adjusted p-value** |
| **RyR2-PDE8A association** | AF vs. Ctl | +0.18 | [-0.47, 0.83] | +0.29 | [-0.79, 1.38] | 0.568 | *N/A* |

**Supplementary Table 1. Statistical summary of experiments.** Quantitative evaluation of live-cell FRET ratio changes, Ca^2+^ spark and wave parameters, and RyR2–PDE8A co-immunoprecipitation in control (Ctl) and atrial fibrillation (AF) myocytes upon total PDE8 inhibition with PF. Estimates, 95% CIs, and Cohen’s *d* effect sizes were derived from Linear Mixed Models (LMM) with cells nested within patients as random effects, adjusted for clinical covariates. Ca^2+^ wave frequency was analyzed using a negative binomial Generalized Linear Mixed Model (GLMM) with an offset term. All p-values were adjusted for multiple testing using False Discovery Rate (FDR) correction (Benjamini–Hochberg). Abbreviations: AF, atrial fibrillation; CI, confidence interval; Co-IP, co-immunoprecipitation; Ctl, control; FDR, false discovery rate; FRET, fluorescence resonance energy transfer; GLMM, generalized linear mixed model; LMM, linear mixed model; PDE8, phosphodiesterase 8; RyR2, ryanodine receptor 2.
