# Supplementary material for: Absence of arrhythmogenic effects in cAMP subcellular compartments supports PDE8 as a target in atrial fibrillation
Source: J Mol Cell Cardiol Plus. 2026 Sep 26;18:100871. doi: 10.1016/j.jmccpl.2026.100871 (PMC13673357; doi:10.1016/j.jmccpl.2026.100871)
Supplement: Supplementary file 3 [file mmc3.docx]

Supplementary Table 2A

| **Assay** | **Ctl N (Patients)** | **Ctl n (Cells)** | **AF N (Patients)** | **AF n (Cells)** |
| --- | --- | --- | --- | --- |
| **Cytosolic cAMP FRET** | 22 | 67 | 20 | 69 |
| **RyR2 cAMP FRET** | 13 | 54 | 9 | 31 |
| **MyBPC cAMP FRET** | 9 | 18 | 8 | 20 |
| **TpnI cAMP FRET** | 14 | 40 | 11 | 20 |
| **Ca^2+^ imaging** | 12 | 13 | 7 | 7 |
| **Co-IP** | 9 | *N/A* | 7 | *N/A* |

Supplementary Table 2B

| **ID** | **Group** | **Chamber** | **Classification** | **FRET Cytosol** | **FRET RyR2** | **FRET MyBPC** | **FRET TpnI** | **Ca^2+^ imaging** | **Co-IP** |
| --- | --- | --- | --- | --- | --- | --- | --- | --- | --- |
| 1 | AF | LA | cAF | X |  |  |  |  |  |
| 2 | CTL | RA | N/A | X |  |  |  |  |  |
| 3 | AF | RA | pAF | X |  |  |  |  |  |
| 4 | CTL | RA | N/A | X |  |  |  |  |  |
| 5 | CTL | RA | N/A | X |  |  |  |  |  |
| 6 | AF | RA | pAF | X |  |  |  |  |  |
| 7 | CTL | RA | N/A | X |  |  |  |  |  |
| 8 | AF | RA | cAF | X |  |  |  |  |  |
| 9 | CTL | RA | N/A | X |  |  |  |  |  |
| 10 | AF | LA | pAF | X |  |  |  |  |  |
| 11 | CTL | RA | N/A | X |  |  |  |  |  |
| 12 | CTL | RA | N/A | X |  |  |  |  |  |
| 13 | AF | RA | cAF | X |  |  |  |  |  |
| 14 | AF | RA | pAF | X |  |  |  |  |  |
| 15 | CTL | RA | N/A | X |  |  |  |  |  |
| 16 | AF | LA | cAF | X |  |  |  |  |  |
| 17 | CTL | RA | N/A | X |  |  |  |  |  |
| 18 | AF | RA | cAF | X |  |  |  |  |  |
| 19 | AF | LA | cAF | X |  |  |  |  |  |
| 20 | CTL | RA | N/A | X |  |  |  |  |  |
| 21 | AF | RA | cAF | X |  |  |  |  |  |
| 22 | CTL | RA | N/A |  | X |  |  |  |  |
| 23 | CTL | RA | N/A |  | X |  |  |  |  |
| 24 | CTL | RA | N/A |  | X |  |  |  |  |
| 25 | CTL | RA | N/A |  | X |  |  |  |  |
| 26 | CTL | RA | N/A |  | X |  |  |  |  |
| 27 | CTL | RA | N/A |  | X |  |  |  |  |
| 28 | AF | RA | pAF |  | X |  |  |  |  |
| 29 | CTL | RA | N/A |  | X |  |  |  |  |
| 30 | CTL | RA | N/A |  | X |  |  |  |  |
| 31 | AF | RA | cAF |  | X |  |  |  |  |
| 32 | AF | RA | cAF |  | X |  |  |  |  |
| 33 | AF | LA | cAF |  | X |  |  |  |  |
| 34 | AF | RA | pAF | X |  |  |  |  |  |
| 35 | AF | RA | cAF |  | X |  |  |  |  |
| 36 | AF | LA | cAF | X | X |  |  |  |  |
| 37 | CTL | RA | N/A |  | X |  |  |  |  |
| 38 | CTL | RA | N/A |  |  | X |  |  |  |
| 39 | CTL | RA | N/A |  |  |  | X |  |  |
| 40 | AF | RA | cAF |  |  | X |  | X |  |
| 41 | CTL | RA | N/A |  |  | X |  |  |  |
| 42 | AF | RA | cAF |  |  | X |  |  |  |
| 43 | AF | RA | cAF |  |  | X |  |  |  |
| 44 | AF | LA | cAF |  |  | X |  |  |  |
| 45 | CTL | RA | N/A |  |  |  |  |  |  |
| 46 | CTL | RA | N/A |  |  | X |  |  |  |
| 47 | CTL | RA | N/A |  |  | X |  |  |  |
| 48 | CTL | RA | N/A |  |  | X |  |  |  |
| 49 | CTL | RA | N/A |  |  | X |  |  |  |
| 50 | AF | RA | cAF |  |  | X |  |  |  |
| 51 | AF | LA | pAF |  |  |  |  | X |  |
| 52 | CTL | RA | N/A |  |  | X |  |  |  |
| 53 | CTL | RA | N/A |  |  | X |  |  |  |
| 54 | AF | LA | cAF |  |  | X |  |  |  |
| 55 | AF | LA | pAF |  |  | X |  |  |  |
| 56 | CTL | RA | N/A |  |  | X |  |  |  |
| 57 | AF | LA | pAF |  |  | X |  | X |  |
| 58 | CTL | LA | N/A |  |  |  |  | X |  |
| 59 | AF | RA | cAF | X |  |  |  |  |  |
| 60 | CTL | RA | N/A | X |  |  |  |  |  |
| 61 | CTL | RA | N/A | X |  |  |  |  |  |
| 62 | CTL | RA | N/A | X |  |  |  |  |  |
| 63 | CTL | RA | N/A | X |  |  |  |  |  |
| 64 | AF | RA | pAF | X |  |  |  |  |  |
| 65 | CTL | RA | N/A | X |  |  |  |  |  |
| 66 | CTL | LA | N/A | X |  |  |  |  |  |
| 67 | CTL | RA | N/A | X |  |  |  |  |  |
| 68 | CTL | LA | N/A | X |  |  |  |  |  |
| 69 | CTL | RA | N/A | X |  |  |  |  |  |
| 70 | AF | LA | cAF | X |  |  |  |  |  |
| 71 | AF | RA | pAF | X |  |  |  |  |  |
| 72 | CTL | LA | N/A | X |  |  |  |  |  |
| 73 | AF | RA | cAF | X |  |  |  |  |  |
| 74 | CTL | RA | N/A | X |  |  |  |  |  |
| 75 | CTL | RA | N/A | X |  |  |  |  |  |
| 76 | AF | RA | cAF | X | X |  |  |  |  |
| 77 | AF | LA | cAF | X |  |  |  |  |  |
| 78 | CTL | RA | N/A |  | X |  |  |  |  |
| 79 | CTL | RA | N/A |  | X |  |  |  |  |
| 80 | CTL | RA | N/A |  | X |  |  |  |  |
| 81 | CTL | RA | N/A |  | X |  |  |  |  |
| 82 | AF | RA | cAF |  | X |  |  |  |  |
| 83 | AF | RA | cAF |  | X |  |  |  |  |
| 84 | CTL | RA | N/A |  |  |  | X |  |  |
| 85 | CTL | RA | N/A |  |  |  | X |  |  |
| 86 | AF | LA | pAF |  |  |  | X |  |  |
| 87 | AF | LA | pAF |  |  |  | X |  |  |
| 88 | AF | RA | cAF |  |  |  | X |  |  |
| 89 | CTL | RA | N/A |  |  |  | X |  |  |
| 90 | CTL | RA | N/A |  |  |  | X |  |  |
| 91 | CTL | RA | N/A |  |  |  | X |  |  |
| 92 | CTL | RA | N/A |  |  |  | X |  |  |
| 93 | AF | RA | pAF |  |  |  | X |  |  |
| 94 | AF | LA | pAF |  |  |  | X |  |  |
| 95 | AF | RA | pAF |  |  |  | X |  |  |
| 96 | CTL | RA | N/A |  |  |  | X |  |  |
| 97 | CTL | RA | N/A |  |  |  | X |  |  |
| 98 | AF | RA | cAF |  |  |  | X |  |  |
| 99 | AF | RA | cAF |  |  |  | X |  |  |
| 100 | AF | LA | pAF |  |  |  | X |  |  |
| 101 | CTL | RA | N/A |  |  |  | X |  |  |
| 102 | CTL | RA | N/A |  |  |  | X |  |  |
| 103 | AF | LA | cAF |  |  |  | X |  |  |
| 104 | CTL | RA | N/A |  |  |  | X |  |  |
| 105 | CTL | RA | N/A |  |  |  | X |  |  |
| 106 | CTL | RA | N/A |  |  |  | X |  |  |
| 107 | AF | LA | pAF |  |  |  | X |  |  |
| 108 | AF | LA | pAF |  |  |  | X |  |  |
| 109 | CTL | RA | N/A |  |  |  | X |  |  |
| 110 | AF | LA | cAF |  |  |  | X |  |  |
| 111 | CTL | RA | N/A |  |  |  |  | X |  |
| 112 | CTL | RA | N/A |  |  |  |  | X |  |
| 113 | CTL | RA | N/A |  |  |  |  | X |  |
| 114 | AF | RA | cAF |  |  |  |  | X |  |
| 115 | CTL | RA | N/A |  |  |  |  | X |  |
| 116 | CTL | RA | N/A |  |  |  |  | X |  |
| 117 | CTL | RA | N/A |  |  |  |  | X |  |
| 118 | CTL | RA | N/A |  |  |  |  | X |  |
| 119 | CTL | RA | N/A |  |  |  |  | X |  |
| 120 | CTL | RA | N/A |  |  |  |  | X |  |
| 121 | CTL | RA | N/A |  |  |  |  | X |  |
| 122 | AF | RA | pAF |  |  |  |  | X |  |
| 123 | AF | RA | pAF |  |  |  |  | X |  |
| 124 | CTL | RA | N/A |  |  |  |  | X |  |
| 125 | CTL | RA | N/A |  |  |  |  |  | X |
| 126 | CTL | RA | N/A |  |  |  |  |  | X |
| 127 | CTL | LA | N/A |  |  |  |  |  | X |
| 128 | AF | RA | cAF |  |  |  |  |  | X |
| 129 | AF | RA | cAF |  |  |  |  |  | X |
| 130 | AF | RA | cAF |  |  |  |  |  | X |
| 131 | AF | RA | cAF |  |  |  |  |  | X |
| 132 | AF | RA | cAF |  |  |  |  |  | X |
| 133 | CTL | RA | N/A |  |  |  |  |  | X |
| 134 | AF | RA | cAF |  |  |  |  |  | X |
| 135 | AF | RA | cAF |  |  |  |  |  | X |
| 136 | CTL | RA | N/A |  |  |  |  |  | X |
| 137 | CTL | RA | N/A |  |  |  |  |  | X |
| 138 | CTL | RA | N/A |  |  |  |  |  | X |
| 139 | CTL | RA | N/A |  |  |  |  |  | X |
| 140 | CTL | RA | N/A |  |  |  |  |  | X |
| 141 | AF | RA | cAF |  |  |  |  |  | X |

**Supplementary Table 2. Sample size allocation, clinical profiles, and tissue fate breakdown across experimental assays.** (A) Summary of cell (n) and patient (N) sample sizes contributing to each experimental sub-assay (FRET live-cell imaging, confocal Ca^2+^ imaging, and Co-IP) in control (Ctl) and atrial fibrillation (AF) cohorts. (B) Individual patient breakdown detailing clinical group, atrial chamber origin (right vs. left atrium), AF classification and specific experimental assay allocation. Abbreviations: AF, atrial fibrillation; cAF, chronic atrial fibrillation; Co-IP, co-immunoprecipitation; Ctl, control; FRET, fluorescence resonance energy transfer; LA, left atrium; N, patient count; n, cell count; N/A, not applicable; pAF, paroxysmal atrial fibrillation; RA, right atrium.
