# Supplementary material for: Absence of arrhythmogenic effects in cAMP subcellular compartments supports PDE8 as a target in atrial fibrillation
Source: J Mol Cell Cardiol Plus. 2026 Sep 26;18:100871. doi: 10.1016/j.jmccpl.2026.100871 (PMC13673357; doi:10.1016/j.jmccpl.2026.100871)
Supplement: Supplementary file 4 [file mmc4.docx]

| **Parameter** | **Minimum Documented Pathological Shift** | **Equivalence Margin** | **PF Effect in AF (Estimate / Upper 95% CI)** |
| --- | --- | --- | --- |
| **Spark Density** | +114% (Hove-Madsen et al., *Circulation* 2004) | +57.0% | +2.7% / +10.9% |
| **Spark Amplitude** | +40% (Nolla-Colomer et al. *Acta Physiologica* 2021) | +20.0% | +13.4% / +23.8% (p = 0.234) |
| **Spark FWHM** | +23% (Tarifa et al., *JACC BTS* 2023) | +11.5% | -2.5% / +3.5% |
| **Spark FDHM / Tau** | +25% (Casabella-Ramón et al., *Biomedicines* 2022) | +12.5% | -2.4% / +3.6% |
| **Wave Frequency** | +1.7 waves/min (Hove-Madsen et al., *Circulation* 2004) | +0.85 waves/min | -1.10 / -0.38 waves/min (p = 0.036) |
| **Wave Amplitude** | +88.8% in ITI / +116% to >15 mV (Tarifa et al., JACC BTS 2023, Schlotthauer and Bers, *Circulation Research*, 2000) | +44.4% to +58.0% | +34.7% / +64.5% (p = 0.072) |

**Supplementary Table 3. Objective equivalence safety analysis of total PDE8 inhibition in AF myocytes.** Functional equivalence evaluation of selective PDE8 inhibition (30 nM PF) on spontaneous Ca^2+^ release dynamics in human AF atrial myocytes. Point estimates and upper 95% CIs were compared against pre-specified objective safety margins (Δ) derived using the half-pathological shift (Δ=Minimum Documented Pathological Shift/2). P-values were derived from LMM or GLMM models with FDR adjustment. AF, atrial fibrillation; CI, confidence interval; FDR, false discovery rate; GLMM, generalized linear mixed model; LMM, linear mixed model; PDE8, phosphodiesterase 8; RyR2, ryanodine receptor 2.
